# Epigenetic Regulation of Cardiomyocyte Differentiation from Embryonic and Induced Pluripotent Stem Cells

**DOI:** 10.3390/ijms22168599

**Published:** 2021-08-10

**Authors:** Yong-Jin Kim, Amin Tamadon, Yoon-Young Kim, Byeong-Cheol Kang, Seung-Yup Ku

**Affiliations:** 1 Department of Obstetrics and Gynecology, Korea University College of Medicine, Seoul 08308, Korea; zinigo@korea.ac.kr; 2 Department of Marine Stem Cell and Tissue Engineering, Bushehr University of Medical Sciences, Bushehr 14174, Iran; amintamaddon@yahoo.com; 3 Department of Obstetrics and Gynecology, Seoul National University College of Medicine, Seoul 03080, Korea; yoonykim@snu.ac.kr; 4 Biomedical Research Institute, Seoul National University Hospital, Seoul 03080, Korea; zinigo@daum.net; 5 Institute of Reproductive Medicine and Population, Medical Research Center, Seoul National University, Seoul 03080, Korea

**Keywords:** epigenetic markers, cardiomyocyte, proliferation, differentiation, induced pluripotent stem cell, embryonic stem cell

## Abstract

With the intent to achieve the best modalities for myocardial cell therapy, different cell types are being evaluated as potent sources for differentiation into cardiomyocytes. Embryonic stem cells and induced pluripotent stem cells have great potential for future progress in the treatment of myocardial diseases. We reviewed aspects of epigenetic mechanisms that play a role in the differentiation of these cells into cardiomyocytes. Cardiomyocytes proliferate during fetal life, and after birth, they undergo permanent terminal differentiation. Upregulation of cardiac-specific genes in adults induces hypertrophy due to terminal differentiation. The repression or expression of these genes is controlled by chromatin structural and epigenetic changes. However, few studies have reviewed and analyzed the epigenetic aspects of the differentiation of embryonic stem cells and induced pluripotent stem cells into cardiac lineage cells. In this review, we focus on the current knowledge of epigenetic regulation of cardiomyocyte proliferation and differentiation from embryonic and induced pluripotent stem cells through histone modification and microRNAs, the maintenance of pluripotency, and its alteration during cardiac lineage differentiation.

## 1. Introduction

During fetal development, the proliferation of cardiomyocytes increases the fetal heart size, but after birth, proliferation is limited and terminal differentiation of the cells results in hypertrophy [1,2,3]. Upregulation of cardiac-specific genes in adults and exit of the cardiomyocytes from the permanent cell cycle causes terminal differentiation in adult cardiomyocytes [1]. The proliferation of adult cardiomyocytes is blocked by silencing the *E2F* genes (encoding a family of transcription factors) that regulate G2/M (mitosis control) and cytokinesis. Epigenetic mechanisms mediate cell cycle gene silencing and cardiac-specific gene upregulation in adults [4].

Regeneration of myocardial tissue in the infarcted wall for the treatment of cardiomyocyte loss is a potential treatment strategy. To achieve this goal, studies are being conducted on pluripotent cells that can differentiate into cardiomyocytes, such as embryonic stem cells and induced pluripotent stem cells. The best stem cell candidate for cell-based therapy of myocardial diseases in regenerative medicine should have two important characteristics: the ability to override immunological rejection and plasticity to differentiate into desired cardiovascular cells.

Non-proliferative characteristics of adult cardiomyocytes and the difficulty of mesenchymal stem cells to differentiate into cardiomyocytes make pluripotent stem cells the best candidate for the production of human cardiomyocytes. Human embryonic stem cells were the first type of pluripotent stem cells to be used in cell therapy for differentiation into cardiomyocytes [5]. However, the most important concerns regarding the use of embryonic stem cells for tissue engineering are the immunogenicity potential and ethical limitations of the use of human embryonic stem cells [6]. These concerns, however, do not exist in the case of induced pluripotent stem cells, which are produced by nuclear reprogramming of somatic cells and can be used as a source for cardiomyocyte production for therapeutic purposes [7]. Direct injection of embryonic stem cells into the heart increases the risk of teratoma formation; therefore, it is necessary to differentiate them into cardiomyocytes prior to implantation [8].

Currently, there are three established methods for differentiating pluripotent stem cells into cardiomyocytes [9]. The first method involves the coculturing of pluripotent stem cells with mouse visceral endoderm-like (*end-2*) stromal cells or culturing in the *end-2*-conditioned medium. The differentiation mechanism is poorly understood in the *end-2*-based method, but it is simpler and inexpensive compared to the embryoid body-based method [10]. Moreover, due to the addition of a MAPK inhibitor, the cardiomyocyte yield efficiency of this method is higher than that of the embryoid body-based method [11]. The second method is the use of embryoid bodies in suspension. Chemical and physical circumstances that mimic early embryonic development together activate molecular pathways for the differentiation of pluripotent stem cells into cardiomyocytes. Small, spherical aggregates of pluripotent stem cells form embryoid bodies. The formation of such embryoid bodies depends on the protocol used; when cultured, 70–97% of these embryoid bodies differentiate into cardiomyocytes [12,13]. Fully defined culture conditions are the most important advantages of this method (Table 1). The disadvantages of this method include the difference in the number of beating cardiomyocytes between different embryoid bodies, low yield efficiency, and immature phenotype of cardiomyocytes [14]. The third method, which was recently developed, involves the addition of small molecules and growth factors into pluripotent stem cell culture medium [9,15]. The earlier studies of a two-dimensional monolayer differentiation were compared with the recent studies of the three-dimensional culture based on the formation of embryoid bodies and spheroids based on the formation of embryoid body and spheroid [16,17]. The advantages of this method include a large scalable quantity of cell differentiation, production of more matured cardiomyocytes, higher cardiomyocyte yields (85–95%), and cost reduction as a result of fewer media components [18].

Nuclear reprogramming via epigenetic modifications in cells for the specification and differentiation of different cell types is a complex process, which is performed by regulating the chromatin structure [31,32]. In induced pluripotent stem cell biology, epigenetic and chromatin modifications are critical, and are reversible and dynamic processes [33]. Several epigenetic factors play important roles in cardiomyocyte differentiation via transcription factors and signaling pathways. These epigenetic factors or regulators include modifications of histone, adenosine triphosphate (ATP)-dependent chromatin remodeling complexes, DNA methylation, and microRNAs (miR) [34,35]. They affect the suppression or expression of a gene by changing the availability of DNA sequences for DNA-binding proteins, inhibiting translation, or cleaving the complementary target messenger RNAs [36]. These alterations result from the modification of DNA-histone covalent interactions, which leads to an increase or decrease in the accessibility of DNA by loosening or tightening the chromatin, respectively, and by post-transcriptional regulation of gene expression. Epigenetic regulation of cardiomyocyte development from embryonic stem cells and induced pluripotent stem cells is the focus of this review.

## 2. The Different Types of Stem Cells: Embryonic Stem Cells and Induced Pluripotent Stem Cells

**Figure 1:**
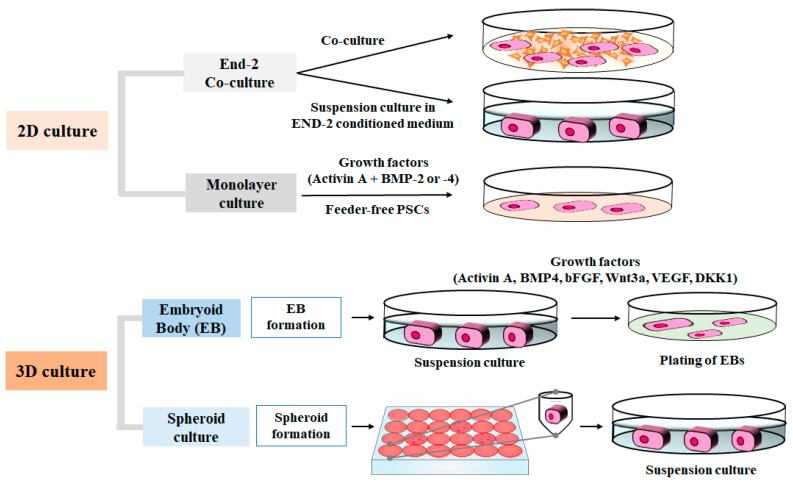
Differentiation induction into cardiomyocytes from pluripotent stem cells (PSCs) using two-dimensional (2D) or three-dimensional (3D) culture.

Protocols to induce cardiac differentiation in human pluripotent stem cells have been previously developed in some studies (Figure 1). The characteristics of embryonic stem cells are different from that of induced pluripotent stem cells. The inherent plasticity of embryonic stem cells could be a potential advantage in their application in regenerative medicine. Moreover, somatic cell nuclear transfer using embryonic stem cells can produce pluripotent stem cells that have the patient’s nuclear genome that can differentiate into cardiomyocytes and repair heart damage. Although this strategy has been used in studies involving animal cardiac repair [37,38], there are various limitations to its applicability in humans, including low efficiency of somatic nuclear transfer, insufficient pluripotency of produced lines, abnormalities encountered in cloned cells, high cost, and the ethical debate surrounding the need for super-ovulated volunteers.

Somatic cells can be reprogrammed into induced pluripotent stem cells via retroviral transduction of *OCT4*, *SOX2*, *KLF4*, and *c-MYC* [7,39,40,41], plasmid transfection without using *c-Myc* [42], using recombinant proteins [43], adenovirus vectors [44], the PiggyBac transposon system [45], cell and transgene-free embryonic stem cell protein extracts [46], or Sendai virus vectors [47]. Induced pluripotent stem cells can differentiate into all three germ lineages. Deriving from autologous sources could be the most valuable aspect of induced pluripotent stem cells. Further, induced pluripotent stem cells are syngeneic. However, there are several limitations to its applicability in humans, including the low efficiency of deriving induced pluripotent stem cells, the long process for therapeutic purposes, and the tendency to form teratomas [48].

**Figure 2:**
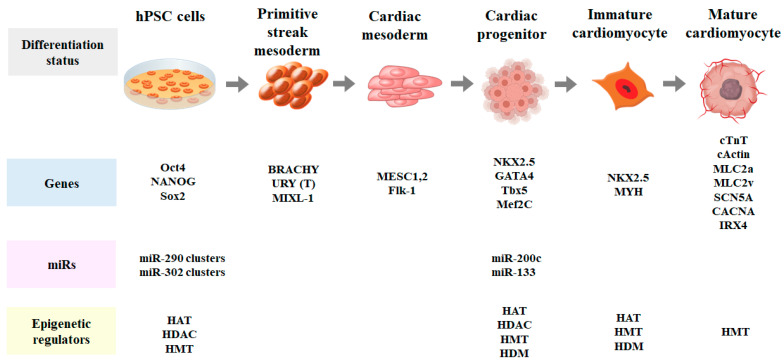
Differentiation of pluripotent stem cells (PSCs) into cardiomyocytes according to stage-specific gene expression and epigenetic regulation.

The epigenetic mechanisms responsible for the induction of pluripotency in somatic cells have not been fully characterized. Mechanistic insights into the reprogramming and retention of induced pluripotency of these cells are crucial for their efficient clinical application. Modifications in the epigenetic makeup of a cell can directly affect gene repression or expression. Epigenetics of embryonic stem cells and induced pluripotent stem cells are extremely complex. To be pluripotent (continuous proliferation, undifferentiation, and differentiation into a particular cell lineage), these cells should have the following three important characteristics: (1) their epigenetic code is continuous and active; (2) genes in the undifferentiated cells remain active, and those in the developing cells are repressed; and (3) they have sufficiently loosened chromatins once they start to differentiate. Modifications of epigenetic enzymes and factors play important roles in all these steps [49,50,51,52,53,54,55,56,57] (Figure 2).

## 3. Epigenetic Regulation of Gene Expression and Silencing

**Figure 3:**
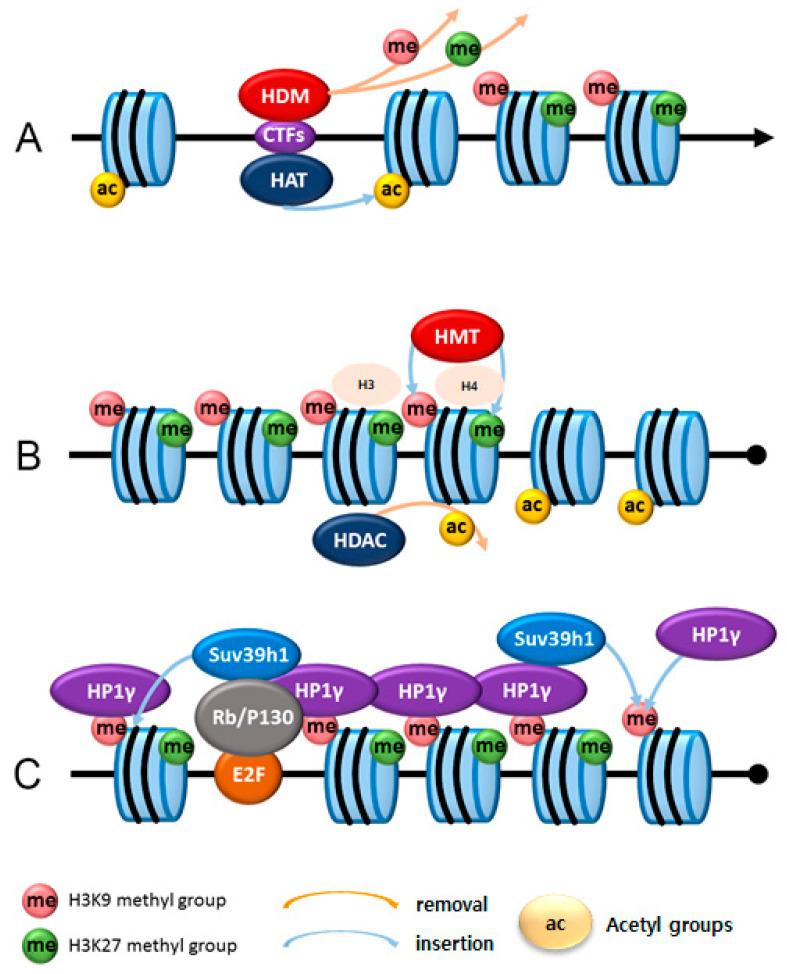
Model of epigenetic gene regulation in cardiomyocytes. (**A**) Activation of cardiac-specific genes: cardiac-specific transcription factors (CTFs) activate cardiac-specific genes by recruiting transferring of acetyl groups to histone H3 and/or H4 histone using acetyltransferases (HAT). Also, they recruit histone demethylases (HDM) to remove silencing methyl marks from H3K9 and H3K27. (**B**) Silencing of non-cardiac genes: histone deacetylases (HDACs) remove acetyl groups (AC) from H3 and/or H4, and histone methyltransferases (HMT) put methyl groups on H3K9 and H3K27 to suppress and silence non-cardiac genes. (**C**) By recruiting HP1γ to E2F responsive promoters, Rb directly promotes permanent cell cycle exit that has undergone methylation of H3K9 (modified from [93]).

Epigenetic changes regulate gene expression and silencing without changes in a DNA sequence. This is enabled by several modification mechanisms, such as histone protein modifications (methylation, acetylation, phosphorylation, sumoylation, ubiquitination, deamination, ribosylation, and proline isomerization), DNA modifications (DNA methylation), modifications using adenosine triphosphate (ATP)-dependent chromatin remodeling complexes, and microRNAs [31] (Table 2). The activation of cell-cycle inhibitors and cardiac-specific genes and repression of cell cycle progression and non-cardiac genes are critical for cardiac differentiation [58,59] (Figure 3).

αMHC, α-myosin heavy chain; αSA, α-sarcomeric actin; ANP, atrial natriuretic peptide; BNP, brain natriuretic peptide; CBP, cAMP response element-binding protein; CDK, cyclin-dependent kinase; Eed, embryonic ectoderm development; Ezh, catalytic subunit enhancer of Zeste; GATA4, critical transcription factor for cardiac development; Hand2, heart- and neural crest derivatives-expressed protein 2; HAT, histone acetyltransferases; HDAC, histone deacetylases; HDM, histone demethylase; HMT, histone methyltransferase; Hopx, homeodomain-only protein; Irx4, iroquois homeobox 4; Kcnip2, Kv channel-interacting protein 2; Mef2, myocyte-specific enhancer factor 2; miR, microRNA; MOF, males absent on the first; PRC2, polycomb repressive complex 2; PTIP, PAX interacting protein 1; RbAp46/48, retinoblastoma protein-associated protein 46/48; skNAC, skeletal nascent polypeptide-associated complex alpha; SRF, serum response factor; Suz12, suppressor of Zeste 12; TBX5, T-box transcription factor 5; UTX, ubiquitously transcribed tetratricopeptide repeat, X chromosome.

### 3.1. Histone Modifications

Gene transcription is affected by several histone modifications such as acetylation, methylation, ubiquitination, pho-phorylation, and sumoylation that occur on lysine and arginine residues of histone tails. Based on our present knowledge of the N-terminus of histones, there are eight acetylate lysine positions, H3K9, H3K14, H3K18, H3K23, H4K5, H4K8, H4K12, and H4K16. H3 and H4 acetylation by histone acetyltransferase and deacetylation by histone deacetylase regulates gene activation and silencing, respectively.

Moreover, six lysine residues of histones can be methylated: H3K4, H3K9, H3K27, H3K36, H3K79, and H4K20. Depending on which amino acids in the histones are methylated, and the number of attached methyl groups, they can either increase or decrease the transcription of DNA. Methylation of H3K4 and H3K27 was found to discriminate between genes that are expressed, poised for expression, or stably repressed; therefore, they could reflect the state and lineage potential of embryonic stem cells [53]. Methylation of H3K36 facilitates gene annotation by marking primary coding and noncoding transcripts [53]. H3K9 and H4K20 methylation are detected in active, telomeric, and satellite long-terminal repeats. Active chromatin has methylated H3K4, whereas inactive chromatin is marked by methylation of H3K9 [94].

Histone acetyltransferases, histone deacetylases, histone methyltransferases, and histone demethylases control these post-translational modifications. Moreover, transcription factors have access to euchromatin, the looser form of chromatin that promotes gene expression, whereas the dense structure, heterochromatin, prevents gene expression [95].

Histone methyltransferases add methyl groups to lysine and arginine residues, while histone demethylases remove methyl groups. Histone methylation forms heterochromatin, and histone acetylation forms euchromatin. Mono, di, or trimethylation of and types of amino acid residues in the histone affect transcriptional activation (for example, by methylation of H3K4, H3K36, and H3K79) or gene repression (for example, by methylation of H3K9 and H3K27) [31]. The trimethylation of H3K9 by recruiting heterochromatin protein 1 (HP1) induces heterochromatin stability [96]. Moreover, trimethylation of H3K27 represses and poises genes [88,97].

### 3.2. Histone Acetyltransferases

p300 histone acetyltransferase activity is expressed in the embryonic myocardium and is required for cardiac development [98,99]. p300 enhances the expression of the α-myosin heavy chain (αMHC) and α-sarcomeric actin (αSA) [60,61]. It also interacts with GATA4 (a transcription factor critical for cardiac development), homeobox protein Nkx-2.5 (a transcription factor critical for regulating tissue differentiation and determining the patterns of temporal and spatial development), and myocyte-specific enhancer factor 2C (MEF2C is involved in cardiac morphogenesis, myogenesis, and vascular development) [62,63,64]. ‘Males-absent on the first’ (MOF) protein, another histone acetyltransferase, plays a critical role in the down-regulation of cardiac hypertrophy in mice and human cardiomyopathies [65]. cAMP response element-binding protein (CBP) is a histone acetyltransferase expressed in the embryonic heart; however, its deficiency does not affect heart formation [66,67]. GCN5, a histone acetyltransferase, plays a role in in vitro cardiac differentiation [68].

### 3.3. Histone Deacetylases

Inhibition of histone deacetylase undergoes acetylation of H3 and H4 and differentiation of cardiomyocytes in vitro [64,100]. Cardiac-specific deletion of histone deacetylase 1 and 2 genes causes neonatal mortality, dilated cardiomyopathy, and cardiac arrhythmias [101]. The interaction of histone deacetylase 2 and HOPX (homeodomain-only protein) limits cardiomyocyte proliferation via GATA4 deacetylation and decreases its transcriptional activity [69,70]. Histone deacetylase 3 suppresses TBX5 activity [102]. Cardiac-specific deletion of histone deacetylase 3 in mice intensifies cardiac hypertrophy [103]. In contrast, overexpression of cardiac histone deacetylase 3 intensified cardiac hyperplasia by the suppression of cyclin-dependent kinase inhibitor 1 (p21^cip1^), 1B (p27^kip1^), 1C (p57^kip2^), 2C (p18^inc4c^), and 2B (p15^inc4b^) without causing hypertrophy [71]. Histone deacetylase 4 prevents cardiac hypertrophy by suppressing *Mef2* [72,73]. The cardiac development functions of histone deacetylase 5 and histone deacetylase 9 overlap and involve the suppression of *Mef2*, and a simultaneous lack of both causes cardiomyocyte abnormality, thin-walled myocardium, and ventricular septal defects [74,75].

Histone deacetylases are recruited by binding of *Hey* proteins close to transcription start sites, leading to deacetylation of histones, condensation of chromatin, and repression of target genes. In cardiomyocytes, the binding of cardiac activators recruits histone acetylases, thereby counteracting *Hey* proteins [104].

Acetylation of H3K56 is associated with the transcriptional activation of pluripotent genes in embryonic stem cells [105]. The NAD-dependent histone deacetylase SIRT6 targets acetylated H3K56 in mouse embryonic stem cells [106]. SIRT6 directly regulates the expression of the core pluripotent genes, *OCT4*, *SOX2*, and *NANOG*, via deacetylation of H3K56, which in turn controls embryonic stem cell differentiation through Tet-mediated oxidation of 5-methylcytosine (5mC) into 5-hydroxymethylcytosine (5hmC) [76].

### 3.4. Histone Methyltransferases

Histone methylation has an important role in regulating cardiac development [78,82,88,107,108,109,110]. De novo mutations in genes that modify H3K4 and H3K27 cause congenital heart disease [111]. PAX interacting protein 1 (*PTIP*), an H3K4 histone methyltransferase subunit, regulates the expression of genes involved in electrical conduction in the heart, such as Kv channel-interacting protein 2 (*KCNIP2*) [77]. Cardiac-specific knockout of *PTIP* altered the regulation of sodium and calcium handling. H3K4 histone methyltransferase does not affect myosin heavy chain beta (*MHC-β*) and atrial natriuretic peptide (*ANP*), which are genes associated with cardiac hypertrophy. SMYD1, a muscle-specific transcription activator, methylates H3K4 in vitro [79,112,113]. SMYD1 plays an efficient role in cardiomyocyte maturation and development of the right ventricle by interacting with skeletal nascent polypeptide-associated complex alpha (skNAC) via upregulation of heart- and neural crest derivatives-expressed protein 2 (HAND2) and iroquois homeobox 4 (IRX4) [78,79,80]. However, the relationship between the suppression of *SYMD1* expression and histone methyltransferase activity in cardiac development is not clear [113,114]. The interaction of *SYMD1* with sarcomere proteins plays a role in myosin protein methylation [114,115]. Moreover, *SYMD1* interacts with histone deacetylases as a transcriptional repressor [78,81]. SMYD2 is a neonatal cardiomyocyte histone methyltransferase of H3K4 and H3K36, and its deficiency can be compensated by other histone methyltransferases [81]. Another histone methyltransferase is Wolf-WHSC1, which mono-, di-, and tri-methylates H3K36. Patients with Wolf–Hirschhorn syndrome have a WHSC1 deletion, which causes congenital cardiac defects [116]. WHSC1 deficiency leads to ventricular and atrial septal defects [107]. Occupation of *Nkx2.5* target genes with *Nkx2.5* and WHSC1 interaction via tri-methylation of H3K36 leads to transcriptional repression [82].

During embryonic stem cell differentiation, polycomb repressive complex 2 (PRC2), a histone methyltransferase complex, activates and occupies target genes *OCT4*, *SOX2*, and *NANOG* in order to maintain pluripotency [51]. PRC2 is recruited by methylated H3K27 [94]; the silence-stage-specific gene *EZH1* mediates the methylation of H3K27 and complements *EZH2* in maintaining stem cell identity and executing pluripotency [83,84,117]. The H3K27 methyltransferase *EZH2* plays an important role in the regulation of gene expression and is related to heart development [118,119]. In female mammalian somatic cells, methylated H3K27 is prominent in the inactivated X-chromosome [94]. An increase in trimethylated H3K27 induces stable recruitment of PRC2 and leads to the differentiation of cardiac progenitor cells into cardiomyocytes [118]. Trimethylated H3K4 fully activates promoters, while H3K4 dimethylation correlates with the basal transcription-permissive state [120]. A pattern of monomethylated H3K4 deposition at the transcription start site of a select group of genes precedes transcriptional activation, acquisition of trimethylated H3K4, and recruitment of RNA polymerase II phosphorylated at serine 5 (RNAP) [59]. This preactivation is important for genes that are not regulated by polycomb complexes.

Embryonic ectoderm development (Eed), retinoblastoma protein-associated protein 46/48 (RBAP46/48), catalytic subunit enhancer of Zeste 1 (EZH1/EZH2), and suppressor of Zeste 12 (SUZ12) are four components of PRC2 [85]. *EZH1* and *EZH2* are predominantly expressed in adult and embryonic hearts [4] and stabilize cardiac differentiation by gene silencing [118,119]. Moreover, *EZH2* binds to and methylates GATA4 and reduces its interaction with p300 and its transcriptional activity, and suppresses α-myosin heavy chain expression in embryonic cardiomyocytes [121]. In addition, G9a and GLP, which are major mono- and di-methyltransferases of H3K9 in cardiomyocytes, play a role in non-cardiac gene silencing during cardiac differentiation [86]. During cardiac differentiation, tri-methylation of H3K9, mediated by *Suv39h1*, regulates cell cycle exit [4]. Therefore, suppressive marks such as di- and trimethylated H3K9 and trimethylated H3K27, which suppress transcriptional activity, lead to cell cycle exit and non-cardiac gene silencing.

### 3.5. Histone Demethylases

Histone methylation is known to be irreversible because the half-life of histones is approximately equal to the half-life of methylated histone [122]. However, the discovery of histone demethylases that remove methyl groups from histones suggests a novel cellular regulatory process. KDM4a is a histone demethylase of trimethylated H3K9 and tri-methylated H3K36 [123]. KDM4a is upregulated and enriched in the atrial natriuretic peptide ANP and brain natriuretic peptide (BNP) promoters [73,87].

The jumonji family proteins have histone demethylase activity and demethylate mono-, di-, and tri-methylation and play an essential role in the development of cardiomyocytes. Jumonji is encoded by the *JARID2* gene and consists of a DNA-binding domain, an AT-rich interaction domain (ARID), and two conserved domains (*JmjN* and *JmjC*) [124]. The JmjC domain is essential for histone demethylation [124,125]. Another member of this family, JMJD6, is a histone demethylase of H3 and H4 arginine, which plays an important role in cardiomyocyte development [89].

Ubiquitously transcribed tetratricopeptide repeat gene on the X chromosome (UTX) is another JMJC protein and an H3K27 demethylase that is encoded by the X chromosome [126,127,128]. UTX plays an important role in the differentiation of embryonic stem cells into cardiomyocytes and the expression of cardiac-specific genes (*ANP*, *MLC2*, and *a-CA*) by demethylation of trimethylated H3K27 [88]. The interaction of UTX with *TFS*, *NKX2.5*, *TBX5*, *GATA4*, serum response factor (SRF), and *Brg1*-associated factor BAF60C promotes cardiac-specific gene activation [88]. In addition, UTX demethylates H3K4 for cardiac enhancer activation [88].

### 3.6. DNA Methylation

DNA methylation is an essential epigenetic mediator for regulating cell development, which is a reversible process critical for embryonic stem cell differentiation [129]. DNA methyltransferase enzymes (DNMT1, DNMT3a, and DNMT3b) attach a methyl group to 50-CpG-30 dinucleotides. DNA methylation occurs at the fifth carbon position in the cytosine residue. The formation of CG dinucleotides establishes a repressed chromatin state and inhibits gene expression [130,131]. DNA methylation plays a role in X-chromosome inactivation, cell differentiation, changing chromatin structure, tumorigenesis, genomic imprinting, tissue-specific gene expression, and induction of pluripotency in somatic cells [130,132]. During the process of demethylation of genes that are involved in the induction of pluripotency in somatic cells and reprogramming them into induced pluripotent stem cells, a cytidine deaminase molecule, AID, plays a key role [133]. The Fe^2+^- and α-ketoglutarate-dependent dioxygenases, and TET enzymes (TET1, TET2, and TET3) catalyze DNA methylation [134,135]. These enzymes revert the methylation status of DNA by successive oxidation of 5mC into 5hmC, 5-carboxy cytosine (5caC), and 5-formyl cytosine (5fC), which are intermediates in an active DNA demethylation mechanism [136,137]. Embryonic stem cell pluripotency is maintained by increasing the levels of 5hmC, TET1, and TET2 [138,139,140]. During the differentiation stage, TET1 and TET2 expression decreases, which leads to repression of pluripotent genes and activation of developmental genes [135,141,142,143,144].

Bivalent domains consist of two near regions: large regions of methylated H3K27 and small regions of methylated H3K4, which were found to silence and equilibrate developmental genes in embryonic stem cells [49]. To induce lineage differentiation of induced pluripotent stem cells into cardiomyocytes, certain small molecules are used to manipulate epigenetic regulators. They include BIX01294 as a histone methyltransferase inhibitor [145], RG108 and 5-azacytidine as DNA methyltransferase inhibitors [146,147], and valproic acid, a histone deacetylase inhibitor [148].

### 3.7. ATP-Dependent Chromatin Remodeling Complexes

ATP-dependent chromatin remodelers use the energy of ATP hydrolysis to disrupt or alter the histone-DNA association, thus providing DNA accessibility [149] via the repositioning of nucleosomes (sliding, twisting, or looping). They include four families, of which the switching-defective and sucrose non-fermenting families are the ones studied more extensively with respect to cardiomyocyte development [149]. The Brahma-related gene 1 (*Brg1*)/Brahma (Brm)-associated factor complex interacts with several cardiac transcription factors, including NKX2-5, GATA4, TBX5, and TBX20 [150,151,152]. BRG1 promotes cardiomyocyte proliferation through BMP10 stimulation, activates the β-myosin heavy chain in the fetal cardiomyocytes, and represses the α-myosin heavy chain in adult cardiomyocytes [153]. Overexpression of TBX5, GATA4, and the BRG1/BRM-associated factor subunit BAF60C promotes differentiation of non-cardiac mesoderm into cardiac tissue [154].

### 3.8. MicroRNAs

**Figure 4:**
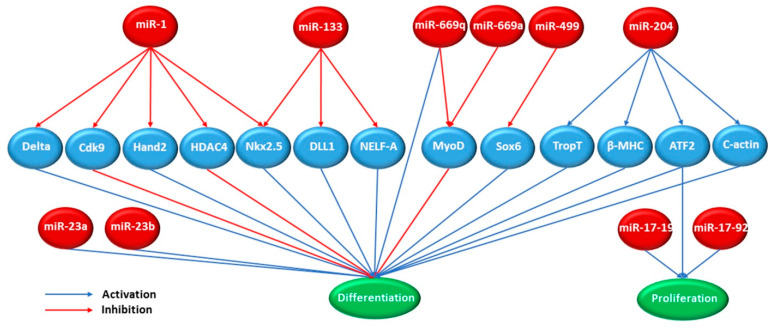
Various miR subtypes (red circle) role in inhibition (red arrows) and activation (blue arrows) of specific transcription factors (blue circle), which are responsible for differentiation and proliferation of cardiomyocytes (modified from [160]). For example, increased miR-1 induces differentiation of cardiac progenitor cells by repressing the translation of cdk9. Conversely, increased miR-133 inhibits this process by repressing the translation of DLL1, a transcription factor that promotes the expression of cardiac mesoderm genes.

MicroRNAs (miRs) are emerging as key players in the reprogramming and differentiation of induced pluripotent stem cells. miRs are small noncoding RNAs that are transcribed from both the intragenic and intergenic regions. They play critical roles in a variety of different processes, and we are beginning to understand their role in pluripotent cells. The role of miRs in pluripotency has been studied using Dicer-null and DGCR8-null ES cells, which lack mature miRs [90,155,156,157]. A subset of miRs controls the expression of DNA methyltransferases, histone deacetylases, and polycomb group genes [158]. When differentiation of Dicer-null ES cells is induced by embryoid body formation, the cells show only a slight decrease in OCT4 levels and a slight increase in the expression of early differentiation genes [90]. This indicates the role of miRs in the early differentiation of these cells. Additionally, miRs seem to play an important role in pluripotency. Two miRs clusters (clusters 290 and 302) have binding sites on their promoter regions for pluripotency-associated genes. Moreover, members of these clusters are regulated by pluripotency-associated genes such as OCT4, SOX2, and NANOG [92,159]. Expression of pluripotency-associated miRs clusters (miR-520) and inhibition of tissue-specific miRs (let-7) during reprogramming can increase the efficiency of the dedifferentiation process [90]. miR-200c represses the differentiation and maturation of human embryonic stem cells into cardiomyocytes by reducing the mRNA levels of GATA4, SRF, and TBX5 [91]. Figure 4 shows various miRs and their roles in the expression or suppression of genes associated with the differentiation and proliferation of cardiomyocytes.

## 4. Terminal Differentiation of Cardiomyocytes

For cardiac lineage differentiation, inducing signals from other lineages and non-cardiac genes is a prerequisite [161]. The cardiac lineage arises from the lateral plate mesoderm during development [162]; BMP signaling is the main signaling pathway associated with cardiac lineage differentiation. Retinoid signaling, and BMP2 signal neighboring the visceral endoderm, are involved in the specification of the cardiac lineage [163,164]. These specific signaling pathways direct early mesodermal lineage-specific genes, Brachyury [165], and the mesendodermal transcription factor GATA4, which is essential for the activation of the cardiac signaling cascade [166,167].

Heterochromatin condensation is a characteristic feature of fully differentiated cells and shows an irreversible exit from the cell cycle [4,168,169,170]. This feature prevents the accessibility of transcriptional factors to heterochromatic loci [171]. Very low histone acetylation and high trimethylation of H3K9 are characteristics of heterochromatic loci. Embryonic cardiomyocytes have high levels of acetylated H3K9/14, H3K18, and H3K27; however, after they are fully differentiated, the level of acetylated histones decreases and trimethylation of H3K9 and H3K27 increases, which leads to repression of the transcription of related genes [4]. These epigenetic processes are mediated by histone deacetylases, histone methyltransferases, Rb family proteins, and HP1 family proteins.

Among the histone deacetylase family members, histone deacetylase 1 plays an important role in cell types other than cardiomyocytes, including retinal cells and oligodendrocytes [172,173,174]. However, there is little information about the role of histone deacetylases in the heterochromatin assembly of differentiated cardiomyocytes. Histone deacetylase 1 plays a critical role in the differentiation, termination, and downregulation of proliferation-promoting proteins of neural cells in the zebrafish retina [172,173].

Histone methyltransferases such as G9a/GLP, Suv39h1/2 di, and tri methylate H3K9 are essential for suppressing cell proliferation and enabling cell cycle exit [4,168,175]. Dimethylated H3K9 is present in euchromatin and heterochromatin loci, while trimethylated H3K9 is found in the heterochromatin region, indicating their different roles in gene silencing [4]. Trimethylated H3K9 along with retinoblastoma protein and p130 can result in heterochromatin formation in cardiomyocytes. HP1 family proteins (HP1α, -β, and -γ) are recruited by Rb proteins and play an important role in gene silencing by maintaining heterochromatin [168,176,177,178,179]. These proteins are expressed in adult cardiomyocytes. However, their role in the heart largely remains unknown, and their subnuclei localize at different sites. HP1γ stably represses adult cardiomyocyte-gene promoters by binding to G2/M and cytokinesis gene promoters, and the other members of the HP1 family, HP1α and -β, cannot compensate for the loss of function in HP1γ [4]. When Rb/p130 is deleted, HP1γ disassociates from the G2/M and cytokinesis gene promoters. Although trimethylated H3K9 remains intact, it leads to the disruption of heterochromatin and cell cycle re-entry by re-expression of G2/M and cytokinesis. However, the role of trimethylated H3K27 in differentiation termination in adult cardiomyocytes is unclear, but it plays a role in the suppression of E2F-dependent genes [180]. Moreover, trimethylation of H3K27 may play a role during the early stages of heterochromatin formation.

## 5. Conclusions

Here, we summarize the results of the studies that have been performed on epigenetic regulation in cardiac differentiation and development. Several reports on gene manipulation techniques and inhibitors have revealed the importance of epigenetic factors in cardiac development. However, specific target genes and histone modification mechanisms as well as the role of related enzymes in cardiac development, require further investigation.

## Figures and Tables

**Table 1:** Cardiac differentiation protocols from human pluripotent stem cells.

Culture Type	Method	Induction Condition	References
3D	EB formation	RPMI 1640 + human serum albumin, phosphoascorbate, ITS, lipid mix, Y-27632, DS-I-7	[12]
	EB formation	RPMI1640 + B27 + CHIR99021	[13]
	EB formation	KO-DMEM + 20% FBS (+DMSO)	[19]
	EB formation	KO-DMEM + 20% FBS (+DMSO)	[16]
	EB formation	Stempro34 w/Activin A, BMP4, bFGF, VEGF, DKK1	[20]
	EB formation	KO DMEM + 15% FBS w/Wnt3a	[21]
	EB formation or monolayer	LI-APEL + Matrigel w/Activin A, BMP4, bFGF, VEGF, SCF, WNT3A	[22]
	EB formation	Stempro34 w/Activin A, BMP4, bFGF, VEGF, DKK1, TGFβi, BMPi	[23]
	EB formation	Stempro34 w/Activin A, BMP4, bFGF, IWR-1, triiodothyronine	[24]
	EB formation	BPEL w/Activin A, BMP4, CHIR99021, SCF, VEGF	[25]
	EB formation	Stempro34 w/BMP4, Activin A, bFGF, IWP2, VEGF, BMP4, RA, bFGFi, TGFβi	[17]
2D	Coculture with visceral endoderm-like cells (END2)	DMEM + 20% FBS	[10]
	Monolayer	RPMI1640 + B27 + MatriGel w/Activin A, BMP4	[26]
	Monolayer	RPMI1640 + B27 + w/Activin A, BMP2	[15]
	Monolayer	RPMI1640 + B27 w/Activin A, BMP4, bFGF, Noggin, RA/RAi, DKK1	[27]
	Monolayer (matrix sandwich)	RPMI1640 + B27 + Matrigel w/Activin A, BMP4, bFGF	[28]
	Monolayer	RPMI1640 + B27 w/CHIR99021, IWP2	[29]
	Monolayer	CDM3 w/CHIR99021, Wnt-C59	[18]
	Monolayer (multilayer plates)	RPMI1640 + B27 + Fibronectin or collagen type I w/CHIR99021, BMP4, IWR-1	[30]

**Table 2:** Epigenetic histone markers in cardiac-specific gene expression.

Epigenetic Factors		Affected Gene	Cardiac Tissue Effect	References
HAT				
	p300	αMHC and αSA	Development	[60,61]
		GATA4	Development	[62]
		Homeobox protein Nkx-2.5	Differentiation	[63]
		Mef2c	Morphogenesis, myogenesis, and vascular development	[64]
	MOF		Down-regulation of cardiac hypertrophy and cardiomyopathies	[65]
	CBP		Unkown	[66,67]
	Gcn5		Differentiation	[68]
HDAC				
	HDAC1,2	Hopx, GATA4	Proliferation	[69,70]
	HDAC3	Suppression of Mef2 and CDK inhibitor 1, 1B, 1C, 2C, 2B	Lack: increased cardiac hypertrophy Expression: cardiac hyperplasia without hypertrophy	[71]
		Suppression of Tbx5	Cardiac hypertrophy	
	HDAC4	Mef2 suppression	Cardiac hypertrophy prevention	[72,73]
	HDAC5,9	Mef2 suppression	Cardiomyocytes abnormality, thin-walled myocardium, and ventricular septal defects	[74,75]
	SIRT6	Oct4, Sox2 and Nanog	Differentiation	[76]
HMT				
	PTIP	Kcnip2	Electrical conduction	[77]
	Smyd1	skNAC	Muscle-specific transcription activator	[78]
		Hand2	Muscle-specific transcription activator	[79]
		Irx4	Muscle-specific transcription activator	[80]
	Smyd2		Neonatal cardiomyocytes histone methyltransferase	[81]
	Wolf-WHSC1	Nkx2.5	Lack: congenital cardiac defects Activity: transcription repression	[82]
	PRC2	Oct4, Sox2, and Nanog	Maintain pluripotency	[51]
		EZH1	Silencestage-specific gene	[83]
		EZH2	Executing pluripotency	[84]
		Eed	Differentiation	[4]
		RbAp46/48	Differentiation	[85]
		Suz12	Differentiation	[85]
	G9a and GLP		Non-cardiac gene silencing	[86]
	Suv39h1		Cell cycle exit	[4]
HDM				
	KDM4a	ANP	Up-regulation	[73]
		BNP	Up-regulation	[87]
	UTX	ANP, MLC2, and a-CA	Differentiation	[88]
		TFs, Nkx2.5 Tbx5, GATA4, SRF, Brg1-associated factor Baf60c	Increase cardiac-specific genes activation	[88]
	JmjC		Development	[89]
	Jmjd6		Development	[89]
miR				
	let-7		Dedifferentiation	[90]
	miR-200c	GATA4, SRF, and TBX5	Repressed differentiation and maturation	[91]
	302-367 cluster	Nanog, Oct3/4, Sox2, and Rex	Development	[92]
	miR-520		Dedifferentiation	[90]

## References

[1] Ahuja P., Sdek P., MacLellan W.R. (2007). Cardiac myocyte cell cycle control in development, disease, and regeneration. Physiol. Rev..

[2] Mollova M., Bersell K., Walsh S., Savla J., Das L.T., Park S.-Y., Silberstein L.E., dos Remedios C.G., Graham D., Colan S. (2013). Cardiomyocyte proliferation contributes to heart growth in young humans. Proc. Natl. Acad. Sci. USA.

[3] Naqvi N., Li M., Calvert J.W., Tejada T., Lambert J.P., Wu J., Kesteven S.H., Holman S.R., Matsuda T., Lovelock J.D. (2014). A proliferative burst during preadolescence establishes the final cardiomyocyte number. Cell.

[4] Sdek P., Zhao P., Wang Y., Huang C.-J., Ko C.Y., Butler P.C., Weiss J.N., MacLellan W.R. (2011). Rb and p130 control cell cycle gene silencing to maintain the postmitotic phenotype in cardiac myocytes. J. Cell Biol..

[5] Evans M.J., Kaufman M.H. (1981). Establishment in culture of pluripotential cells from mouse embryos. Nature.

[6] McLaren A. (2001). Ethical and social considerations of stem cell research. Nature.

[7] Takahashi K., Tanabe K., Ohnuki M., Narita M., Ichisaka T., Tomoda K., Yamanaka S. (2007). Induction of pluripotent stem cells from adult human fibroblasts by defined factors. Cell.

[8] Bernstein H.S., Srivastava D. (2012). Stem cell therapy for cardiac disease. Pediatric Res..

[9] Mummery C.L., Zhang J., Ng E.S., Elliott D.A., Elefanty A.G., Kamp T.J. (2012). Differentiation of human embryonic stem cells and induced pluripotent stem cells to cardiomyocytes a methods overview. Circ. Res..

[10] Mummery C., Ward-van Oostwaard D., Doevendans P., Spijker R., van den Brink S., Hassink R., Van der Heyden M., Opthof T., Pera M., de la Riviere A.B. (2003). Differentiation of human embryonic stem cells to cardiomyocytes role of coculture with visceral endoderm-like cells. Circulation.

[11] Graichen R., Xu X., Braam S.R., Balakrishnan T., Norfiza S., Sieh S., Soo S.Y., Tham S.C., Mummery C., Colman A. (2008). Enhanced cardiomyogenesis of human embryonic stem cells by a small molecular inhibitor of p38 MAPK. Differentiation.

[12] Breckwoldt K., Letuffe-Brenière D., Mannhardt I., Schulze T., Ulmer B., Werner T., Benzin A., Klampe B., Reinsch M.C., Laufer S. (2017). Differentiation of cardiomyocytes and generation of human engineered heart tissue. Nat. Protoc..

[13] Kempf H., Kropp C., Olmer R., Martin U., Zweigerdt R. (2015). Cardiac differentiation of human pluripotent stem cells in scalable suspension culture. Nat. Protoc..

[14] Kawamura M., Miyagawa S., Miki K., Saito A., Fukushima S., Higuchi T., Kawamura T., Kuratani T., Daimon T., Shimizu T. (2012). Feasibility, safety, and therapeutic efficacy of human induced pluripotent stem cell-derived cardiomyocyte sheets in a porcine ischemic cardiomyopathy model. Circulation.

[15] Kim Y.Y., Ku S.Y., Liu H.C., Cho H.J., Oh S.K., Moon S.Y., Choi Y.M. (2011). Cryopreservation of human embryonic stem cells derived-cardiomyocytes induced by BMP2 in serum-free condition. Reprod. Sci..

[16] Kim Y.Y., Ku S.Y., Jang J., Oh S.K., Kim H.S., Kim S.H., Choi Y.M., Moon S.Y. (2008). Use of long-term cultured embryoid bodies may enhance cardiomyocyte differentiation by BMP2. Yonsei Med. J..

[17] Protze S.I., Liu J., Nussinovitch U., Ohana L., Backx P.H., Gepstein L., Keller G.M. (2017). Sinoatrial node cardiomyocytes derived from human pluripotent cells function as a biological pacemaker. Nat. Biotechnol..

[18] Burridge P.W., Matsa E., Shukla P., Lin Z.C., Churko J.M., Ebert A.D., Lan F., Diecke S., Huber B., Mordwinkin N.M. (2014). Chemically defined generation of human cardiomyocytes. Nat. Methods.

[19] Kehat I., Kenyagin-Karsenti D., Snir M., Segev H., Amit M., Gepstein A., Livne E., Binah O., Itskovitz-Eldor J., Gepstein L. (2001). Human embryonic stem cells can differentiate into myocytes with structural and functional properties of cardiomyocytes. J. Clin. Investig..

[20] Yang L., Soonpaa M.H., Adler E.D., Roepke T.K., Kattman S.J., Kennedy M., Henckaerts E., Bonham K., Abbott G.W., Linden R.M. (2008). Human cardiovascular progenitor cells develop from a KDR plus embryonic-stem-cell-derived population. Nature.

[21] Tran T.H., Wang X., Browne C., Zhang Y., Schinke M., Izumo S., Burcin M. (2009). Wnt3a-induced mesoderm formation and cardiomyogenesis in human embryonic stem cells. Stem Cells.

[22] Elliott D.A., Braam S.R., Koutsis K., Ng E.S., Jenny R., Lagerqvist E.L., Biben C., Hatzistavrou T., Hirst C.E., Yu Q.C. (2011). NKX2-5(eGFP/w) hESCs for isolation of human cardiac progenitors and cardiomyocytes. Nat. Methods.

[23] Kattman S.J., Witty A.D., Gagliardi M., Dubois N.C., Niapour M., Hotta A., Ellis J., Keller G. (2011). Stage-specific optimization of activin/nodal and BMP signaling promotes cardiac differentiation of mouse and human pluripotent stem cell lines. Cell Stem Cell.

[24] Willems E., Spiering S., Davidovics H., Lanier M., Xia Z., Dawson M., Cashman J., Mercola M. (2011). Small-molecule inhibitors of the Wnt pathway potently promote cardiomyocytes from human embryonic stem cell-derived mesoderm. Circ. Res..

[25] Devalla H.D., Schwach V., Ford J.W., Milnes J.T., El-Haou S., Jackson C., Gkatzis K., Elliott D.A., Chuva de Sousa Lopes S.M., Mummery C.L. (2015). Atrial-like cardiomyocytes from human pluripotent stem cells are a robust preclinical model for assessing atrial-selective pharmacology. EMBO Mol. Med..

[26] Laflamme M.A., Chen K.Y., Naumova A.V., Muskheli V., Fugate J.A., Dupras S.K., Reinecke H., Xu C., Hassanipour M., Police S. (2007). Cardiomyocytes derived from human embryonic stem cells in pro-survival factors enhance function of infarcted rat hearts. Nat. Biotechnol..

[27] Zhang Q., Jiang J., Han P., Yuan Q., Zhang J., Zhang X., Xu Y., Cao H., Meng Q., Chen L. (2011). Direct differentiation of atrial and ventricular myocytes from human embryonic stem cells by alternating retinoid signals. Cell Res..

[28] Zhang J., Klos M., Wilson G.F., Herman A.M., Lian X., Raval K.K., Barron M.R., Hou L., Soerens A.G., Yu J. (2012). Extracellular matrix promotes highly efficient cardiac differentiation of human pluripotent stem cells: The matrix sandwich method. Circ. Res..

[29] Lian X., Zhang J., Azarin S.M., Zhu K., Hazeltine L.B., Bao X., Hsiao C., Kamp T.J., Palecek S.P. (2013). Directed cardiomyocyte differentiation from human pluripotent stem cells by modulating Wnt/beta-catenin signaling under fully defined conditions. Nat. Protoc..

[30] Tohyama S., Hattori F., Sano M., Hishiki T., Nagahata Y., Matsuura T., Hashimoto H., Suzuki T., Yamashita H., Satoh Y. (2013). Distinct metabolic flow enables large-scale purification of mouse and human pluripotent stem cell-derived cardiomyocytes. Cell Stem Cell.

[31] Chen T., Dent S.Y.R. (2014). Chromatin modifiers and remodellers: Regulators of cellular differentiation. Nat. Rev. Genet..

[32] Li G., Reinberg D. (2011). Chromatin higher-order structures and gene regulation. Curr. Opin. Genet. Dev..

[33] Kooistra S.M., Helin K. (2012). Molecular mechanisms and potential functions of histone demethylases. Nat. Rev. Mol. Cell Biol..

[34] Paige S.L., Plonowska K., Xu A., Wu S.M. (2015). Molecular regulation of cardiomyocyte differentiation. Circ. Res..

[35] Huang W., Feng Y., Liang J., Yu H., Wang C., Wang B., Wang M., Jiang L., Meng W., Cai W. (2018). Loss of microRNA-128 promotes cardiomyocyte proliferation and heart regeneration. Nat. Commun..

[36] Bartel D.P. (2009). MicroRNAs: Target recognition and regulatory functions. Cell.

[37] Kofidis T., de Bruin J.L., Yamane T., Tanaka M., Lebl D.R., Swijnenburg R.-J., Weissman I.L., Robbins R.C. (2005). Stimulation of paracrine pathways with growth factors enhances embryonic stem cell engraftment and host-specific differentiation in the heart after ischemic myocardial injury. Circulation.

[38] Rajasingh J., Bord E., Hamada H., Lambers E., Qin G., Losordo D.W., Kishore R. (2007). STAT3-dependent mouse embryonic stem cell differentiation into cardiomyocytes: Analysis of molecular signaling and therapeutic efficacy of cardiomyocyte precommitted mES transplantation in a mouse model of myocardial infarction. Circ. Res..

[39] Park I.-H., Zhao R., West J.A., Yabuuchi A., Huo H., Ince T.A., Lerou P.H., Lensch M.W., Daley G.Q. (2008). Reprogramming of human somatic cells to pluripotency with defined factors. Nature.

[40] Takahashi K., Okita K., Nakagawa M., Yamanaka S. (2007). Induction of pluripotent stem cells from fibroblast cultures. Nat. Protoc..

[41] Takahashi K., Yamanaka S. (2006). Induction of pluripotent stem cells from mouse embryonic and adult fibroblast cultures by defined factors. Cell.

[42] Nakagawa M., Koyanagi M., Tanabe K., Takahashi K., Ichisaka T., Aoi T., Okita K., Mochiduki Y., Takizawa N., Yamanaka S. (2008). Generation of induced pluripotent stem cells without Myc from mouse and human fibroblasts. Nat. Biotechnol..

[43] Kim D., Kim C.-H., Moon J.-I., Chung Y.-G., Chang M.-Y., Han B.-S., Ko S., Yang E., Cha K.Y., Lanza R. (2009). Generation of human induced pluripotent stem cells by direct delivery of reprogramming proteins. Cell Stem Cell.

[44] Tashiro K., Kawabata K., Inamura M., Takayama K., Furukawa N., Sakurai F., Katayama K., Hayakawa T., Furue M.K., Mizuguchi H. (2010). Adenovirus vector-mediated efficient transduction into human embryonic and induced pluripotent stem cells. Cell. Reprogramming.

[45] Woltjen K., Michael I.P., Mohseni P., Desai R., Mileikovsky M., Hämäläinen R., Cowling R., Wang W., Liu P., Gertsenstein M. (2009). piggyBac transposition reprograms fibroblasts to induced pluripotent stem cells. Nature.

[46] Rajasingh J., Lambers E., Hamada H., Bord E., Thorne T., Goukassian I., Krishnamurthy P., Rosen K.M., Ahluwalia D., Zhu Y. (2008). Cell-free embryonic stem cell extract-mediated derivation of multipotent stem cells from NIH3T3 fibroblasts for functional and anatomical ischemic tissue repair. Circ. Res..

[47] Ban H., Nishishita N., Fusaki N., Tabata T., Saeki K., Shikamura M., Takada N., Inoue M., Hasegawa M., Kawamata S. (2011). Efficient generation of transgene-free human induced pluripotent stem cells (iPSCs) by temperature-sensitive Sendai virus vectors. Proc. Natl. Acad. Sci. USA.

[48] Stadtfeld M., Hochedlinger K. (2010). Induced pluripotency: History, mechanisms, and applications. Genes Dev..

[49] Bernstein B.E., Mikkelsen T.S., Xie X., Kamal M., Huebert D.J., Cuff J., Fry B., Meissner A., Wernig M., Plath K. (2006). A bivalent chromatin structure marks key developmental genes in embryonic stem cells. Cell.

[50] Holliday R. (2006). Epigenetics: A historical overview. Epigenetics.

[51] Lee T.I., Jenner R.G., Boyer L.A., Guenther M.G., Levine S.S., Kumar R.M., Chevalier B., Johnstone S.E., Cole M.F., Isono K.-I. (2006). Control of developmental regulators by Polycomb in human embryonic stem cells. Cell.

[52] Ma P., Schultz R.M. (2008). Histone deacetylase 1 (HDAC1) regulates histone acetylation, development, and gene expression in preimplantation mouse embryos. Dev. Biol..

[53] Mikkelsen T.S., Ku M., Jaffe D.B., Issac B., Lieberman E., Giannoukos G., Alvarez P., Brockman W., Kim T.-K., Koche R.P. (2007). Genome-wide maps of chromatin state in pluripotent and lineage-committed cells. Nature.

[54] Simon J.A., Kingston R.E. (2009). Mechanisms of polycomb gene silencing: Knowns and unknowns. Nat. Rev. Mol. Cell Biol..

[55] Vakoc C.R., Sachdeva M.M., Wang H., Blobel G.A. (2006). Profile of histone lysine methylation across transcribed mammalian chromatin. Mol. Cell. Biol..

[56] Xi S., Geiman T.M., Briones V., Guang Tao Y., Xu H., Muegge K. (2009). Lsh participates in DNA methylation and silencing of stem cell genes. Stem Cells.

[57] Hajkova P. (2011). Epigenetic reprogramming in the germline: Towards the ground state of the epigenome. Philos. Trans. R. Soc. B Biol. Sci..

[58] Paige S.L., Thomas S., Stoick-Cooper C.L., Wang H., Maves L., Sandstrom R., Pabon L., Reinecke H., Pratt G., Keller G. (2012). A temporal chromatin signature in human embryonic stem cells identifies regulators of cardiac development. Cell.

[59] Wamstad J.A., Alexander J.M., Truty R.M., Shrikumar A., Li F., Eilertson K.E., Ding H., Wylie J.N., Pico A.R., Capra J.A. (2012). Dynamic and coordinated epigenetic regulation of developmental transitions in the cardiac lineage. Cell.

[60] Yao T.-P., Oh S.P., Fuchs M., Zhou N.-D., Ch’ng L.-E., Newsome D., Bronson R.T., Li E., Livingston D.M., Eckner R. (1998). Gene dosage-dependent embryonic development and proliferation defects in mice lacking the transcriptional integrator p300. Cell.

[61] Partanen M., Motoyama J., Hui C.C. (1999). Developmentally regulated expression of the transcriptional cofactors/histone acetyltransferases CBP and p300 during mouse embryogenesis. Int. J. Dev. Biol..

[62] Ma K., Chan J.K.L., Zhu G., Wu Z. (2005). Myocyte enhancer factor 2 acetylation by p300 enhances its DNA binding activity, transcriptional activity, and myogenic differentiation. Mol. Cell. Biol..

[63] Sun H., Yang X., Zhu J., Lv T., Chen Y., Chen G., Zhong L., Li Y., Huang X., Huang G. (2010). Inhibition of p300-HAT results in a reduced histone acetylation and down-regulation of gene expression in cardiac myocytes. Life Sci..

[64] Kawamura T., Ono K., Morimoto T., Wada H., Hirai M., Hidaka K., Morisaki T., Heike T., Nakahata T., Kita T. (2005). Acetylation of GATA-4 is involved in the differentiation of embryonic stem cells into cardiac myocytes. J. Biol. Chem..

[65] Qiao W., Zhang W., Gai Y., Zhao L., Fan J. (2014). The histone acetyltransferase MOF overexpression blunts cardiac hypertrophy by targeting ROS in mice. Biochem. Biophys. Res. Commun..

[66] Tanaka Y., Naruse I., Hongo T., Xu M.-J., Nakahata T., Maekawa T., Ishii S. (2000). Extensive brain hemorrhage and embryonic lethality in a mouse null mutant of CREB-binding protein. Mech. Dev..

[67] Chen G., Zhu J., Lv T., Wu G., Sun H., Huang X., Tian J. (2009). Spatiotemporal expression of histone acetyltransferases, p300 and CBP, in developing embryonic hearts. J. Biomed. Sci..

[68] Li L., Zhu J., Tian J., Liu X., Feng C. (2010). A role for Gcn5 in cardiomyocyte differentiation of rat mesenchymal stem cells. Mol. Cell. Biochem..

[69] Trivedi C.M., Zhu W., Wang Q., Jia C., Kee H.J., Li L., Hannenhalli S., Epstein J.A. (2010). Hopx and Hdac2 interact to modulate Gata4 acetylation and embryonic cardiac myocyte proliferation. Dev. Cell.

[70] Chen F., Kook H., Milewski R., Gitler A.D., Lu M.M., Li J., Nazarian R., Schnepp R., Jen K., Biben C. (2002). Hop is an unusual homeobox gene that modulates cardiac development. Cell.

[71] Trivedi C.M., Lu M.M., Wang Q., Epstein J.A. (2008). Transgenic overexpression of Hdac3 in the heart produces increased postnatal cardiac myocyte proliferation but does not induce hypertrophy. J. Biol. Chem..

[72] Backs J., Worst B.C., Lehmann L.H., Patrick D.M., Jebessa Z., Kreusser M.M., Sun Q., Chen L., Heft C., Katus H.A. (2011). Selective repression of MEF2 activity by PKA-dependent proteolysis of HDAC4. J. Cell Biol..

[73] Hohl M., Wagner M., Reil J.-C., Müller S.-A., Tauchnitz M., Zimmer A.M., Lehmann L.H., Thiel G., Böhm M., Backs J. (2013). HDAC4 controls histone methylation in response to elevated cardiac load. J. Clin. Investig..

[74] Zhang C.L., McKinsey T.A., Chang S., Antos C.L., Hill J.A., Olson E.N. (2002). Class II histone deacetylases act as signal-responsive repressors of cardiac hypertrophy. Cell.

[75] Chang S., McKinsey T.A., Zhang C.L., Richardson J.A., Hill J.A., Olson E.N. (2004). Histone deacetylases 5 and 9 govern responsiveness of the heart to a subset of stress signals and play redundant roles in heart development. Mol. Cell. Biol..

[76] Etchegaray J.-P., Chavez L., Huang Y., Ross K.N., Choi J., Martinez-Pastor B., Walsh R.M., Sommer C.A., Lienhard M., Gladden A. (2015). The histone deacetylase SIRT6 controls embryonic stem cell fate via TET-mediated production of 5-hydroxymethylcytosine. Nat. Cell Biol..

[77] Stein A.B., Jones T.A., Herron T.J., Patel S.R., Day S.M., Noujaim S.F., Milstein M.L., Klos M., Furspan P.B., Jalife J. (2011). Loss of H3K4 methylation destabilizes gene expression patterns and physiological functions in adult murine cardiomyocytes. J. Clin. Investig..

[78] Gottlieb P.D., Pierce S.A., Sims R.J., Yamagishi H., Weihe E.K., Harriss J.V., Maika S.D., Kuziel W.A., King H.L., Olson E.N. (2002). Bop encodes a muscle-restricted protein containing MYND and SET domains and is essential for cardiac differentiation and morphogenesis. Nat. Genet..

[79] Sirinupong N., Brunzelle J., Ye J., Pirzada A., Nico L., Yang Z. (2010). Crystal structure of cardiac-specific histone methyltransferase SmyD1 reveals unusual active site architecture. J. Biol. Chem..

[80] Park C.Y., Pierce S.A., von Drehle M., Ivey K.N., Morgan J.A., Blau H.M., Srivastava D. (2010). skNAC, a Smyd1-interacting transcription factor, is involved in cardiac development and skeletal muscle growth and regeneration. Proc. Natl. Acad. Sci. USA.

[81] Costantini D.L., Arruda E.P., Agarwal P., Kim K.-H., Zhu Y., Zhu W., Lebel M., Cheng C.W., Park C.Y., Pierce S.A. (2005). The homeodomain transcription factor Irx5 establishes the mouse cardiac ventricular repolarization gradient. Cell.

[82] Movassagh M., Choy M.-K., Knowles D.A., Cordeddu L., Haider S., Down T., Siggens L., Vujic A., Simeoni I., Penkett C. (2011). Distinct epigenomic features in end-stage failing human hearts. Circulation.

[83] Boyer L.A., Plath K., Zeitlinger J., Brambrink T., Medeiros L.A., Lee T.I., Levine S.S., Wernig M., Tajonar A., Ray M.K. (2006). Polycomb complexes repress developmental regulators in murine embryonic stem cells. Nature.

[84] Shen X., Liu Y., Hsu Y.-J., Fujiwara Y., Kim J., Mao X., Yuan G.-C., Orkin S.H. (2008). EZH1 mediates methylation on histone H3 lysine 27 and complements EZH2 in maintaining stem cell identity and executing pluripotency. Mol. Cell.

[85] Margueron R.l., Reinberg D. (2011). The Polycomb complex PRC2 and its mark in life. Nature.

[86] Inagawa M., Nakajima K., Makino T., Ogawa S., Kojima M., Ito S., Ikenishi A., Hayashi T., Schwartz R.J., Nakamura K. (2013). Histone H3 lysine 9 methyltransferases, G9a and GLP are essential for cardiac morphogenesis. Mech. Dev..

[87] Zhang Q.-J., Chen H.-Z., Wang L., Liu D.-P., Hill J.A., Liu Z.-P. (2011). The histone trimethyllysine demethylase JMJD2A promotes cardiac hypertrophy in response to hypertrophic stimuli in mice. J. Clin. Investig..

[88] Lee S., Lee J.W., Lee S.-K. (2012). UTX, a histone H3-lysine 27 demethylase, acts as a critical switch to activate the cardiac developmental program. Dev. Cell.

[89] Chang B., Chen Y., Zhao Y., Bruick R.K. (2007). JMJD6 is a histone arginine demethylase. Science.

[90] Mallanna S.K., Rizzino A. (2010). Emerging roles of microRNAs in the control of embryonic stem cells and the generation of induced pluripotent stem cells. Dev. Biol..

[91] Poon E.N., Hao B., Guan D., Jun Li M., Lu J., Yang Y., Wu B., Wu S.C., Webb S.E., Liang Y. (2018). Integrated transcriptomic and regulatory network analyses identify microRNA-200c as a novel repressor of human pluripotent stem cell-derived cardiomyocyte differentiation and maturation. Cardiovasc. Res..

[92] Barroso-delJesus A., Romero-López C., Lucena-Aguilar G., Melen G.J., Sanchez L., Ligero G., Berzal-Herranz A., Menendez P. (2008). Embryonic stem cell-specific miR302-367 cluster: Human gene structure and functional characterization of its core promoter. Mol. Cell. Biol..

[93] Oyama K., El-Nachef D., Zhang Y., Sdek P., MacLellan W.R. (2014). Epigenetic regulation of cardiac myocyte differentiation. Front. Genet..

[94] Lachner M., O’Sullivan R.J., Jenuwein T. (2003). An epigenetic road map for histone lysine methylation. J. Cell Sci..

[95] Johnson A., Wu R., Peetz M., Gygi S.P., Moazed D. (2013). Heterochromatic gene silencing by activator interference and a transcription elongation barrier. J. Biol. Chem..

[96] Canzio D., Liao M., Naber N., Pate E., Larson A., Wu S., Marina D.B., Garcia J.F., Madhani H.D., Cooke R. (2013). A conformational switch in HP1 releases auto-inhibition to drive heterochromatin assembly. Nature.

[97] Rada-Iglesias A., Bajpai R., Swigut T., Brugmann S.A., Flynn R.A., Wysocka J. (2011). A unique chromatin signature uncovers early developmental enhancers in humans. Nature.

[98] Shikama N., Lutz W., Kretzschmar R., Sauter N., Roth J.F., Marino S., Wittwer J., Scheidweiler A., Eckner R. (2003). Essential function of p300 acetyltransferase activity in heart, lung and small intestine formation. EMBO J..

[99] Schueler M., Zhang Q., Schlesinger J., Tönjes M., Sperling S.R. (2012). Dynamics of Srf, p300 and histone modifications during cardiac maturation in mouse. Mol. Biosyst..

[100] Karamboulas C., Swedani A., Ward C., Al-Madhoun A.S., Wilton S., Boisvenue S., Ridgeway A.G., Skerjanc I.S. (2006). HDAC activity regulates entry of mesoderm cells into the cardiac muscle lineage. J. Cell Sci..

[101] Montgomery R.L., Davis C.A., Potthoff M.J., Haberland M., Fielitz J., Qi X., Hill J.A., Richardson J.A., Olson E.N. (2007). Histone deacetylases 1 and 2 redundantly regulate cardiac morphogenesis, growth, and contractility. Genes Dev..

[102] Montgomery R.L., Potthoff M.J., Haberland M., Qi X., Matsuzaki S., Humphries K.M., Richardson J.A., Bassel-Duby R., Olson E.N. (2008). Maintenance of cardiac energy metabolism by histone deacetylase 3 in mice. J. Clin. Investig..

[103] Lewandowski S.L., Janardhan H.P., Smee K.M., Bachman M., Sun Z., Lazar M.A., Trivedi C.M. (2014). Histone deacetylase 3 modulates Tbx5 activity to regulate early cardiogenesis. Hum. Mol. Genet..

[104] Weber D., Heisig J., Kneitz S., Wolf E., Eilers M., Gessler M. (2015). Mechanisms of epigenetic and cell-type specific regulation of Hey target genes in ES cells and cardiomyocytes. J. Mol. Cell. Cardiol..

[105] Xie W., Song C., Young N.L., Sperling A.S., Xu F., Sridharan R., Conway A.E., Garcia B.A., Plath K., Clark A.T. (2009). Histone h3 lysine 56 acetylation is linked to the core transcriptional network in human embryonic stem cells. Mol. Cell.

[106] Yang B., Zwaans B.M.M., Eckersdorff M., Lombard D.B. (2009). The sirtuin SIRT6 deacetylates H3 K56Ac in vivo to promote genomic stability. Cell Cycle.

[107] Nimura K., Ura K., Shiratori H., Ikawa M., Okabe M., Schwartz R.J., Kaneda Y. (2009). A histone H3 lysine 36 trimethyltransferase links Nkx2-5 to Wolf-Hirschhorn syndrome. Nature.

[108] Barski A., Cuddapah S., Cui K., Roh T.-Y., Schones D.E., Wang Z., Wei G., Chepelev I., Zhao K. (2007). High-resolution profiling of histone methylations in the human genome. Cell.

[109] Fujii T., Tsunesumi S.-i., Yamaguchi K., Watanabe S., Furukawa Y. (2011). Smyd3 is required for the development of cardiac and skeletal muscle in zebrafish. PLoS ONE.

[110] Tao Y., Neppl R.L., Huang Z.-P., Chen J., Tang R.-H., Cao R., Zhang Y., Jin S.-W., Wang D.-Z. (2011). The histone methyltransferase Set7/9 promotes myoblast differentiation and myofibril assembly. J. Cell Biol..

[111] Zaidi S., Choi M., Wakimoto H., Ma L., Jiang J., Overton J.D., Romano-Adesman A., Bjornson R.D., Breitbart R.E., Brown K.K. (2013). De novo mutations in histone-modifying genes in congenital heart disease. Nature.

[112] Sims R.J., Weihe E.K., Zhu L., O’Malley S., Harriss J.V., Gottlieb P.D. (2002). m-Bop, a repressor protein essential for cardiogenesis, interacts with skNAC, a heart-and muscle-specific transcription factor. J. Biol. Chem..

[113] Tan X., Rotllant J., Li H., DeDeyne P., Du S.J. (2006). SmyD1, a histone methyltransferase, is required for myofibril organization and muscle contraction in zebrafish embryos. Proc. Natl. Acad. Sci. USA.

[114] Just S., Meder B., Berger I.M., Etard C., Trano N., Patzel E., Hassel D., Marquart S., Dahme T., Vogel B. (2011). The myosin-interacting protein SMYD1 is essential for sarcomere organization. J. Cell Sci..

[115] Li H., Zhong Y., Wang Z., Gao J., Xu J., Chu W., Zhang J., Fang S., Du S.J. (2013). Smyd1b is required for skeletal and cardiac muscle function in zebrafish. Mol. Biol. Cell.

[116] Bergemann A.D., Cole F., Hirschhorn K. (2005). The etiology of Wolf-Hirschhorn syndrome. Trends Genet..

[117] Pasini D., Bracken A.P., Hansen J.B., Capillo M., Helin K. (2007). The polycomb group protein Suz12 is required for embryonic stem cell differentiation. Mol. Cell. Biol..

[118] Delgado-Olguín P., Huang Y., Li X., Christodoulou D., Seidman C.E., Seidman J.G., Tarakhovsky A., Bruneau B.G. (2012). Epigenetic repression of cardiac progenitor gene expression by Ezh2 is required for postnatal cardiac homeostasis. Nat. Genet..

[119] He A., Ma Q., Cao J., von Gise A., Zhou P., Xie H., Zhang B., Hsing M., Christodoulou D.C., Cahan P. (2012). Polycomb repressive complex 2 regulates normal development of the mouse heart. Circ. Res..

[120] Santos-Rosa H., Schneider R., Bannister A.J., Sherriff J., Bernstein B.E., Emre N.C.T., Schreiber S.L., Mellor J., Kouzarides T. (2002). Active genes are tri-methylated at K4 of histone H3. Nature.

[121] He A., Shen X., Ma Q., Cao J., von Gise A., Zhou P., Wang G., Marquez V.E., Orkin S.H., Pu W.T. (2012). PRC2 directly methylates GATA4 and represses its transcriptional activity. Genes Dev..

[122] Pedersen M.T., Helin K. (2010). Histone demethylases in development and disease. Trends Cell Biol.

[123] Whetstine J.R., Nottke A., Lan F., Huarte M., Smolikov S., Chen Z., Spooner E., Li E., Zhang G., Colaiacovo M. (2006). Reversal of histone lysine trimethylation by the JMJD2 family of histone demethylases. Cell.

[124] Takeuchi T., Watanabe Y., Takano-Shimizu T., Kondo S. (2006). Roles of jumonji and jumonji family genes in chromatin regulation and development. Dev. Dyn..

[125] Klose R.J., Kallin E.M., Zhang Y. (2006). JmjC-domain-containing proteins and histone demethylation. Nat. Rev. Genet..

[126] Agger K., Cloos P.A.C., Christensen J., Pasini D., Rose S., Rappsilber J., Issaeva I., Canaani E., Salcini A.E., Helin K. (2007). UTX and JMJD3 are histone H3K27 demethylases involved in HOX gene regulation and development. Nature.

[127] Hong S., Cho Y.-W., Yu L.-R., Yu H., Veenstra T.D., Ge K. (2007). Identification of JmjC domain-containing UTX and JMJD3 as histone H3 lysine 27 demethylases. Proc. Natl. Acad. Sci. USA.

[128] Lan F., Bayliss P.E., Rinn J.L., Whetstine J.R., Wang J.K., Chen S., Iwase S., Alpatov R., Issaeva I., Canaani E. (2007). A histone H3 lysine 27 demethylase regulates animal posterior development. Nature.

[129] Smith Z.D., Meissner A. (2013). DNA methylation: Roles in mammalian development. Nat. Rev. Genet..

[130] Klose R.J., Bird A.P. (2006). Genomic DNA methylation: The mark and its mediators. Trends Biochem. Sci..

[131] Hemberger M., Dean W., Reik W. (2009). Epigenetic dynamics of stem cells and cell lineage commitment: Digging Waddington’s canal. Nat. Rev. Mol. Cell Biol..

[132] Trojer P., Reinberg D. (2006). Histone lysine demethylases and their impact on epigenetics. Cell.

[133] Dudley D.D., Chaudhuri J., Bassing C.H., Alt F.W. (2005). Mechanism and control of V (D) J recombination versus class switch recombination: Similarities and differences. Adv. Immunol..

[134] Iyer L.M., Tahiliani M., Rao A., Aravind L. (2009). Prediction of novel families of enzymes involved in oxidative and other complex modifications of bases in nucleic acids. Cell Cycle.

[135] Tahiliani M., Koh K.P., Shen Y., Pastor W.A., Bandukwala H., Brudno Y., Agarwal S., Iyer L.M., Liu D.R., Aravind L. (2009). Conversion of 5-methylcytosine to 5-hydroxymethylcytosine in mammalian DNA by MLL partner TET1. Science.

[136] He Y.-F., Li B.-Z., Li Z., Liu P., Wang Y., Tang Q., Ding J., Jia Y., Chen Z., Li L. (2011). Tet-mediated formation of 5-carboxylcytosine and its excision by TDG in mammalian DNA. Science.

[137] Ito S., Shen L., Dai Q., Wu S.C., Collins L.B., Swenberg J.A., He C., Zhang Y. (2011). Tet proteins can convert 5-methylcytosine to 5-formylcytosine and 5-carboxylcytosine. Science.

[138] Ficz G., Branco M.R., Seisenberger S., Santos F.T., Krueger F., Hore T.A., Marques C.J., Andrews S., Reik W. (2011). Dynamic regulation of 5-hydroxymethylcytosine in mouse ES cells and during differentiation. Nature.

[139] Williams K., Christensen J., Pedersen M.T., Johansen J.V., Cloos P.A.C., Rappsilber J., Helin K. (2011). TET1 and hydroxymethylcytosine in transcription and DNA methylation fidelity. Nature.

[140] Wu H., D’Alessio A.C., Ito S., Wang Z., Cui K., Zhao K., Sun Y.E., Zhang Y. (2011). Genome-wide analysis of 5-hydroxymethylcytosine distribution reveals its dual function in transcriptional regulation in mouse embryonic stem cells. Genes Dev..

[141] Kriaucionis S., Heintz N. (2009). The nuclear DNA base 5-hydroxymethylcytosine is present in Purkinje neurons and the brain. Science.

[142] Ito S., D’Alessio A.C., Taranova O.V., Hong K., Sowers L.C., Zhang Y. (2010). Role of Tet proteins in 5mC to 5hmC conversion, ES-cell self-renewal and inner cell mass specification. Nature.

[143] Ko M., Huang Y., Jankowska A.M., Pape U.J., Tahiliani M., Bandukwala H.S., An J., Lamperti E.D., Koh K.P., Ganetzky R. (2010). Impaired hydroxylation of 5-methylcytosine in myeloid cancers with mutant TET2. Nature.

[144] Szwagierczak A., Bultmann S., Schmidt C.S., Spada F., Leonhardt H. (2010). Sensitive enzymatic quantification of 5-hydroxymethylcytosine in genomic DNA. Nucleic Acids Res..

[145] Mezentseva N.V., Yang J., Kaur K., Iaffaldano G., Rémond M.C., Eisenberg C.A., Eisenberg L.M. (2012). The histone methyltransferase inhibitor BIX01294 enhances the cardiac potential of bone marrow cells. Stem Cells Dev..

[146] Savickiene J., Treigyte G., Jazdauskaite A., Borutinskaite V.V., Navakauskiene R. (2012). DNA methyltransferase inhibitor RG108 and histone deacetylase inhibitors cooperate to enhance NB4 cell differentiation and E-cadherin re-expression by chromatin remodelling. Cell Biol. Int..

[147] Qian Q., Qian H., Zhang X., Zhu W., Yan Y., Ye S., Peng X., Li W., Xu Z., Sun L. (2011). 5-Azacytidine induces cardiac differentiation of human umbilical cord-derived mesenchymal stem cells by activating extracellular regulated kinase. Stem Cells Dev..

[148] Minami I., Yamada K., Otsuji T.G., Yamamoto T., Shen Y., Otsuka S., Kadota S., Morone N., Barve M., Asai Y. (2012). A small molecule that promotes cardiac differentiation of human pluripotent stem cells under defined, cytokine-and xeno-free conditions. Cell Rep..

[149] Chang C.-P., Bruneau B.G. (2012). Epigenetics and cardiovascular development. Annu. Rev. Physiol..

[150] Bevilacqua A., Willis M.S., Bultman S.J. (2014). SWI/SNF chromatin-remodeling complexes in cardiovascular development and disease. Cardiovasc. Pathol..

[151] Lickert H., Takeuchi J.K., von Both I., Walls J.R., McAuliffe F., Adamson S.L., Henkelman R.M., Wrana J.L., Rossant J., Bruneau B.G. (2004). Baf60c is essential for function of BAF chromatin remodelling complexes in heart development. Nature.

[152] Takeuchi J.K., Lou X., Alexander J.M., Sugizaki H., Delgado-Olguín P., Holloway A.K., Mori A.D., Wylie J.N., Munson C., Zhu Y. (2011). Chromatin remodelling complex dosage modulates transcription factor function in heart development. Nat. Commun..

[153] Hang C.T., Yang J., Han P., Cheng H.-L., Shang C., Ashley E., Zhou B., Chang C.-P. (2010). Chromatin regulation by Brg1 underlies heart muscle development and disease. Nature.

[154] Takeuchi J.K., Bruneau B.G. (2009). Directed transdifferentiation of mouse mesoderm to heart tissue by defined factors. Nature.

[155] Lee Y., Jeon K., Lee J.T., Kim S., Kim V.N. (2002). MicroRNA maturation: Stepwise processing and subcellular localization. EMBO J..

[156] Lewis B.P., Burge C.B., Bartel D.P. (2005). Conserved seed pairing, often flanked by adenosines, indicates that thousands of human genes are microRNA targets. Cell.

[157] Zeng Y., Cullen B.R. (2003). Sequence requirements for micro RNA processing and function in human cells. RNA.

[158] Sato F., Tsuchiya S., Meltzer S.J., Shimizu K. (2011). MicroRNAs and epigenetics. FEBS J..

[159] Card D.A.G., Hebbar P.B., Li L., Trotter K.W., Komatsu Y., Mishina Y., Archer T.K. (2008). Oct4/Sox2-regulated miR-302 targets cyclin D1 in human embryonic stem cells. Mol. Cell. Biol..

[160] Purvis N., Bahn A., Katare R. (2015). The Role of MicroRNAs in Cardiac Stem Cells. Stem Cells Int..

[161] Stottmann R.W., Choi M., Mishina Y., Meyers E.N., Klingensmith J. (2004). BMP receptor IA is required in mammalian neural crest cells for development of the cardiac outflow tract and ventricular myocardium. Development.

[162] Jiang Z., Zhu L., Hu L., Slesnick T.C., Pautler R.G., Justice M.J., Belmont J.W. (2013). Zic3 is required in the extra-cardiac perinodal region of the lateral plate mesoderm for left-right patterning and heart development. Hum. Mol. Genet..

[163] Zhang H., Bradley A. (1996). Mice deficient for BMP2 are nonviable and have defects in amnion/chorion and cardiac development. Development.

[164] Xavier-Neto J., Sousa Costa A.M., Figueira A.C., Caiaffa C.D., Amaral F.N., Peres L.M., da Silva B.S., Santos L.N., Moise A.R., Castillo H.A. (2015). Signaling through retinoic acid receptors in cardiac development: Doing the right things at the right times. Biochim. Biophys. Acta.

[165] Beisaw A., Tsaytler P., Koch F., Schmitz S.U., Melissari M.T., Senft A.D., Wittler L., Pennimpede T., Macura K., Herrmann B.G. (2018). BRACHYURY directs histone acetylation to target loci during mesoderm development. EMBO Rep..

[166] Maitra M., Schluterman M.K., Nichols H.A., Richardson J.A., Lo C.W., Srivastava D., Garg V. (2009). Interaction of Gata4 and Gata6 with Tbx5 is critical for normal cardiac development. Dev. Biol..

[167] Holtzinger A., Rosenfeld G.E., Evans T. (2010). Gata4 directs development of cardiac-inducing endoderm from ES cells. Dev. Biol..

[168] Narita M., Nuñez S., Heard E., Narita M., Lin A.W., Hearn S.A., Spector D.L., Hannon G.J., Lowe S.W. (2003). Rb-mediated heterochromatin formation and silencing of E2F target genes during cellular senescence. Cell.

[169] Brero A., Easwaran H.P., Nowak D., Grunewald I., Cremer T., Leonhardt H., Cardoso M.C. (2005). Methyl CpG-binding proteins induce large-scale chromatin reorganization during terminal differentiation. J. Cell Biol..

[170] Sdek P., Oyama K., Angelis E., Chan S.S., Schenke-Layland K., MacLellan W.R. (2013). Epigenetic regulation of myogenic gene expression by heterochromatin protein 1 alpha. PLoS ONE.

[171] Grewal S.I.S., Jia S. (2007). Heterochromatin revisited. Nat. Rev. Genet..

[172] Stadler J.A., Shkumatava A., Norton W.H.J., Rau M.J., Geisler R., Fischer S., Neumann C.J. (2005). Histone deacetylase 1 is required for cell cycle exit and differentiation in the zebrafish retina. Dev. Dyn..

[173] Yamaguchi M., Tonou-Fujimori N., Komori A., Maeda R., Nojima Y., Li H., Okamoto H., Masai I. (2005). Histone deacetylase 1 regulates retinal neurogenesis in zebrafish by suppressing Wnt and Notch signaling pathways. Development.

[174] Ye F., Chen Y., Hoang T., Montgomery R.L., Zhao X.-h., Bu H., Hu T., Taketo M.M., van Es J.H., Clevers H. (2009). HDAC1 and HDAC2 regulate oligodendrocyte differentiation by disrupting the β-catenin–TCF interaction. Nat. Neurosci..

[175] Kotake Y., Cao R., Viatour P., Sage J., Zhang Y., Xiong Y. (2007). pRB family proteins are required for H3K27 trimethylation and Polycomb repression complexes binding to and silencing p16INK4a tumor suppressor gene. Genes Dev..

[176] Nielsen S.J., Schneider R., Bauer U.-M., Bannister A.J., Morrison A., O’Carroll D., Firestein R., Cleary M., Jenuwein T., Herrera R.E. (2001). Rb targets histone H3 methylation and HP1 to promoters. Nature.

[177] Daniel J.A., Pray-Grant M.G., Grant P.A. (2005). Effector proteins for methylated histones: An expanding family. Cell Cycle.

[178] James T.C., Elgin S.C. (1986). Identification of a nonhistone chromosomal protein associated with heterochromatin in Drosophila melanogaster and its gene. Mol. Cell. Biol..

[179] Kellum R. (2003). HP1 complexes and heterochromatin assembly. Protein Complexes that Modify Chromatin.

[180] Blais A., van Oevelen C.J.C., Margueron R.l., Acosta-Alvear D., Dynlacht B.D. (2007). Retinoblastoma tumor suppressor protein-dependent methylation of histone H3 lysine 27 is associated with irreversible cell cycle exit. J. Cell Biol..

